# Mesenchymal Stem Cell Therapy Modulates the Inflammatory Response in Experimental Traumatic Brain Injury

**DOI:** 10.1155/2011/564089

**Published:** 2011-06-09

**Authors:** Layla T. Galindo, Thais R. M. Filippo, Patricia Semedo, Carolina B. Ariza, Caroline M. Moreira, Niels O. S. Camara, Marimelia A. Porcionatto

**Affiliations:** ^1^Departamento de Bioquímica, Universidade Federal de São Paulo, Rua Três de Maio 100, 04044-020 São Paulo, SP, Brazil; ^2^Departamento de Imunologia, Universidade de São Paulo, 05508-000 São Paulo, SP, Brazil

## Abstract

Therapy with mesenchymal stem cells (MSCs) has showed to be promising due to its immunomodulatory function. Traumatic brain injury (TBI) triggers immune response and release of inflammatory mediators, mainly cytokines, by glial cells creating a hostile microenvironment for endogenous neural stem cells (NSCs). We investigated the effects of factors secreted by MSCs on NSC *in vitro* and analyzed cytokines expression *in vitro* in a TBI model. Our *in vitro* results show that MSC-secreted factors increase NSC proliferation and induce higher expression of GFAP, indicating a tendency toward differentiation into astrocytes. *In vivo* experiments showed that MSC injection at an acute model of brain injury diminishes a broad profile of cytokines in the tissue, suggesting that MSC-secreted factors may modulate the inflammation at the injury site, which may be of interest to the development of a favorable microenvironment for endogenous NSC and consequently to repair the injured tissue.

## 1. Background

For many years, the central nervous system (CNS) was considered an immunologically privileged site, due to its particular and limited immune response, different from other systems [1]. It is now clear that CNS is connected to the immune and endocrine systems and has a local inflammatory response, which can contribute to the pathophysiology of acute and chronic brain diseases [2, 3]. Acute neurodegenerative conditions such as traumatic brain injury (TBI) are characterized by severe neuronal loss [4]. TBI breaks the impermeability of the blood brain barrier, which allows the invasion of immune cells and activation of glial cells, mainly microglia and astrocytes, key cells for the immune response within CNS, triggering the secretion of inflammatory mediators by those cells [5, 6]. 

Inflammatory molecules are important in healthy nervous tissue and their expression is promptly upregulated when an injury occurs [3]. Cytokines are the main molecules in neuroinflammatory response and are critical for its regulation, exerting a variety of activities [7, 8]. Microglia and astrocytes release several cytokines, such as IL-1*α*, IL-1*β*, IL-6, TNF-*α*, and TGF-*β*, chemokines, and prostaglandins, which increase the blood brain barrier permeability and facilitate the invasion of peripheral immune cells, inducing the secretion of toxic molecules [3, 7]. 

In the adult brain, chemoattractants, including the chemokine CXCL12 produced at the injury site induce neuroblasts originated by neural stem cells (NSC) at neurogenic niches, such as the subventricular zone (SVZ), to migrate towards the injury in order to regenerate the nervous tissue [9–11]. Although neuroblasts leave the neurogenic niche arrive at the injury site, only a few survive due to the injury inflammatory microenvironment that is thought to be unfavorable to neuroblast survival and differentiation into mature neurons [12]. The limited CNS capacity of regeneration and the complex environment created by TBI, starting at the acute phase, followed by a secondary phase that continues for weeks after the primary insult [13] lead researchers to try to find potential therapies using neuroprotective and anti-inflammatory drugs [14–18]. 

Cell-based therapy using adult-derived mesenchymal stem cells (MSC) have been tested in several disease models [19–24]. It is well accepted that transplantation of MSC, particularly those derived from bone marrow, promotes tissue repair via secreted soluble factors that enhance tissue regeneration, stimulate proliferation, migration, and differentiation of endogenous stem-like progenitors found in most tissues, as well as by decreasing inflammatory and immune reactions and apoptosis [25, 26]. The ability of such cells to modify tissue microenvironment through its trophic influence may contribute more significantly than their capacity for transdifferentiation in effecting tissue repair. 

In order to better understand the paracrine effects of MSC transplanted into an experimental brain injury site on local cytokine production we have used a TBI model in mice, transplanted bone marrow-derived MSC into the lesion, and analyzed the production of cytokines. We also investigated the effects of soluble factors secreted by MSC on neural stem cells survival, proliferation, and differentiation *in vitro*. Our results show a decrease in the production of inflammatory cytokines 24 hours after MSC transplantation. We also show that soluble factors secreted by MSC increase proliferation and modulate gene expression of neural stem cells *in vitro*, increasing the expression of GFAP, a marker of glial cells. 

## 2. Material and Methods

### 2.1. Animals

Adult (6-week-old for MSC and NSC isolation, 12-week-old for TBI model) C57BL/6 male mice were used in this study. The animals were maintained under standard conditions (light/dark cycle 12 h/12 h, constant room temperature at 22 ± 2°C, food, and water *ad libitum*). All the experimental procedures were conducted according to international regulation and were approved by the Committee for Ethics in Research of Universidade Federal de São Paulo (approval no. 1367/08). 

### 2.2. Isolation, Expansion, and Characterization of MSC

After euthanasia in CO_2_ chamber, bone marrow was obtained from femur of adult mice. The femora were removed, cleaned, and placed in DMEM low glucose (*Dulbecco's Modified Eagle's Medium*, GibcoBRL, San Francisco, USA). Inside the laminar flow, the epiphyses were cut and bone marrow was flushed by DMEM into a culture dish using a syringe. The volume obtained from each femur was treated separately. Bone marrow was suspended and centrifuged for 5 minutes at 400 ×g. The supernatant was discarded and the cell pellet derived from each femur was suspended in DMEM low glucose containing 10% fetal bovine serum (FBS, Cultilab, Campinas, SP, Brazil), 1% glutamine (Sigma Chemical Co., St. Louis, MO, USA), and 1% penicillin/streptomycin (GibcoBRL) and cultured in six-well culture dishes (Corning Incorporated, NY, USA) at a volume of 3.5 mL/well. Cultures were kept in a humidified 5% CO_2_ incubator at 37°C for 72 hours, when nonadherent cells were removed by changing the medium. Culture medium was changed every 3 days until adherent cells (MSC) reached 70–80% confluence, then cells were washed with 0.03% PBS/EDTA, detached with 0.1% trypsin (Cultilab), and replated in a 1 : 2 split ratio. Differentiation potential was checked by culturing the MSC under favorable conditions for adipogenic and osteogenic differentiation, as previously described [27]. To obtain the MSC conditioned medium (MSC-CM), confluent cultures (passages 5–10) were kept in DMEM low glucose containing 0.5% FBS for 96 hours. After this period, conditioned medium was centrifuged for 5 minutes at 400 ×g, the supernatant was collected, immediately frozen and kept at −80°C until its use. 

### 2.3. Isolation of Adult NSC and Neurosphere Formation

Adult NSC were obtained from adult C57BL/6 male mice SVZ. After euthanasia by cervical dislocation, brain was removed, the SVZ dissected under a microscope, and the cells maintained in DMEM high glucose (GibcoBRL). After sedimentation, supernatant was discarded, and cells were dissociated by incubation with 0.1% trypsin/EDTA during 5 minutes at 37°C. FBS was added to stop trypsin action, cells were centrifuged for 5 minutes at 400 ×g, and supernatant was removed. Isolated cells were then suspended in DMEM/F12 7 : 3 (v/v) (GibcoBRL), supplemented with 2% B27 (GibcoBRL), 20 ng/mL EGF (Sigma), 20 ng/mL FGF2 (R&D, Minneapolis, MN, USA), 1% penicillin/streptomycin (GibcoBRL), and 5 *μ*g/mL heparin (Sigma). After mechanical dissociation, cells were plated on a polyHEMA (Sigma) precoated 75 cm^2^ flask at a density of 2.4 × 10^7^ cells/flask or on a 24-well plate at a density of 2 × 10^6^ cells/well. Neurosphere formation takes up to 14–21 days to occur, and during that time culture medium was changed every 4-5 days by centrifugation for 5 minutes at 400 ×g. Half volume of the medium was replaced by fresh medium. Neurospheres were cultured with or without MSC-CM for 2 days to evaluate NSC viability and for 4 days to evaluate NSC survival and differentiation. 

### 2.4. MTT Assay

The MTT (3-(4,5-dimethylthiazol-yl)2,5-diphenyltetrazolium bromide, Sigma) assay was performed to evaluate NSC viability cultured for 48 hours in MSC-CM. At the end of this period, neurospheres were centrifuged for 5 minutes at 400 ×g, supernatant was discarded, and 270 *μ*L of culture medium and 30 *μ*L of MTT solution (5 mg/L) were added to each well and incubated for 3 hours at 37°C. After this period, MTT solution was aspirated and the formazan reaction products were dissolved by the addition of 180 *μ*l of dimethyl sulfoxide (DMSO, MP Biomedicals, Solon, Ohio, USA) to each well. The plate was shaken for 15 minutes, the content was transferred to a 96-well plate, and the optical density was read at 540 nm on an ELISA plate reader (Labsystems Multiskan MS, Helsinki, Finland). 

### 2.5. Proliferation Assay

*In vitro *adult NSC proliferation was measured by flow cytometry (FACSCalibur System, BD Biosciences, San Jose, CA, USA, using the software *Windows Multiple Document Interface Flow Cytometry Application*, WinMDI Version 2.9, The Scripps Research Institute, San Diego, CA, USA), after BrdU incorporation. Adult NSC were cultured as neurospheres and maintained for 4 days in MSC-CM or in control medium (DMEM/F12 7 : 3 v/v, supplemented with 2% B27, 20 ng/mL EGF, 20 ng/mL FGF2, 1% penicillin/streptomycin, and 5 *μ*g/mL heparin) at 37°C. BrdU was added during the last 24 hours before analysis. Neurospheres were dissociated with 0.1% trypsin for 5 minutes at 37°C, fixed by 1% paraformaldehyde (PFA) in 0.1 M PBS, incubated in 2 M HCl for 1 hour at 37°C, washed with 0.1 M PBS, blocked, and permeabilized with 10% FBS and 0.1% TritonX-100 in 0.1 M PBS. Cells were incubated with anti-BrdU (rat IgG 1 : 300, Accurate Chemical and Scientific Corporation, Westbury, NY, USA) for 1 hour and washed with 0.1M PBS. Cells were then incubated with secondary antibody (Alexa Fluor488-conjugated goat anti-rat IgG, 1 : 300, Molecular Probes, Carlsbad, CA, USA) for 40 minutes. 

### 2.6. Injury to Mice Primary Motor Cortex and MSC Transplantation

Adult C57BL/6 male mice were submitted to surgery under anesthesia with intraperitoneal administration of xilasine (32 mg/kg)/ketamine chloridrate (66 mg/kg) (Dopalen, Vetbrands, São Paulo, Brazil). Traumatic injury to mice motor cortex was performed according to previously described protocol [28, 29]. Briefly, a metal needle was chilled using isopentane on dry ice and was inserted 4 times, during 30 seconds each, into the motor cortex (stereotaxic coordinates from bregma: AP +0.198 mm; ML +0.175 mm; DV −0.15 mm [30]). MSC (1 × 10^5^ cells in 3 *μ*L) were injected at the injury site using a Hamilton syringe under the same stereotaxic coordinates and the same anesthesia, immediately after the injury. Control consisted of animals without injury and animals that were submitted to the injury but did not receive MSC (injury, no treatment). Brain tissue was collected 24 hours (acute injury) or 30 days (chronic injury) after MSC injection. Mice were anesthetized by i.p. injection of sodium thiopental (Tiopentax, Cristália, São Paulo, Brazil), brain was removed from skull, the motor cortex was dissected and total RNA was extracted by Trizol Reagent (Life Technologies, Carlsbad, CA, USA). Animal's blood was collected by cardiac puncture, centrifuged to separate the serum, which was immediately frozen in dry ice and kept at −80°C until use. 

### 2.7. qPCR

Total RNA was isolated from dissected motor cortex or neurospheres using Trizol Reagent (Life Technologies), and RNA concentration was determined using NanoDrop ND-1000 (Thermo Fisher Scientific, Wilmington, MA, USA). Reverse transcriptase reactions were performed with ImProm-II Reverse Transcription System (Promega, Madison, WI, USA) using 2 *μ*g of total RNA. 

Brain tissue samples qPCR was performed using ABI PRISM 7500 Sequence Detector, using Sequence Detection Software 1.9 for analysis (Applied Biosystems, Foster City, CA, USA), using TaqMan probes (Applied Biosystems) for HPRT (Mm00446968_m1; endogenous control gene), IL-4 (Mm00445259_m1), IL-6 (Mm00446190_m1), IL-10 (Mm00439614_m1), and TNF*α* (Mm00443258_m1) according to the manufacturer's recommendations. Values are expressed relatively to control RNA obtained from motor cortex from mice that were not submitted to surgery. 

Neurosphere samples qPCR was performed using Brilliant II SYBR Green QPCR Master Mix (Stratagene, La Jolla, CA, EUA) using Mx3000P QPCR System using MxPro qPCR Software for analysis (Stratagene). Primers sequences were GAPDH (endogenous control gene; sense 5′-AAGAAGGTGGTGAAGCAGGCATCT-3′; antisense 5′-ACCCTGTTGCTGTAGCCGTATTCA-3′), GFAP (sense 5′-CTCAGTACGAGGCAGTGGCC-3′; antisense 5′-CGGGAAGCAACGTCTGTGA-3′), nestin (sense 5′-AGCAACTGGCACACCTCAAG-3′; antisense 5′-GGTGTCTGCAAGCGAAAGTTC-3′), GAP-43 (sense 5′-AAGGAGGAGCCTAAACAAGCCGAT-3′; antisense 5′-TAGGTTTGGCTTCGTCTACAGCGT-3′), and SOX2 (sense 5′-ATCCCATCCAAATTAACGCA-3′; antisense 5′-GAAGCGCCTAACGTACCACT-3′). Values are expressed relatively to RNA from neurospheres cultured in regular medium (control). 

The quantification of the target genes was normalized by an endogenous control gene (HPRT for brain tissue samples and GAPDH for neurosphere samples). The threshold cycle (Ct) for the target gene and the Ct for the internal control were determined for each sample, run in triplicates. The relative expression of mRNA was calculated by 2^−ΔΔCt^ method [31]. 

### 2.8. Bio-Plex

In order to analyze alterations in cytokines expression in mice serum after TBI, a Bio-Plex assay was performed. Custom Bio-Rad (Bio-Rad laboratories, Hercules, CA, USA). Bio-Plex cytokine analysis kits were used together with the Bio-Plex system array reader according to the manufacturer's directions. The following cytokines were quantified: CCL2 (MCP-1), CCL3 (MIP-1*α*), CCL4 (MIP-1*β*), CCL5 (RANTES), eotaxin (CCL11), CXCL1 (KC), CXCL2 (MIP-2), CXCL5 (LIX), CXCL9 (MIG), CXCL10 (IP-10), G-CSF, GM-CSF, INF-*γ*, IL-1*β*, IL-4, IL-5, IL-6, IL-10, IL-13, IL-17, LIF, TNF-*α*, and VEGF. Samples were run in triplicate for each assay. The assay was read on the Bio-Plex suspension array system, and the data were analyzed using Bio-Plex Manager software version 4.0. Standard curves ranged from 10,000 to 3,2 pg/mL. 

### 2.9. Statistical Analysis

Data are expressed as mean ± SEM. Differences between groups were evaluated by one-way analysis of variance (ANOVA) followed by Tukey's posttest by the use of GraphPad Prism software version 4.0 (GraphPad Software, La Jolla, CA, USA). Statistical significance was set at *P* < .05. 

## 3. Results

### 3.1. MSC Transplantation Modulates Cytokines Expression after Acute Brain Injury

**Figure 1:**
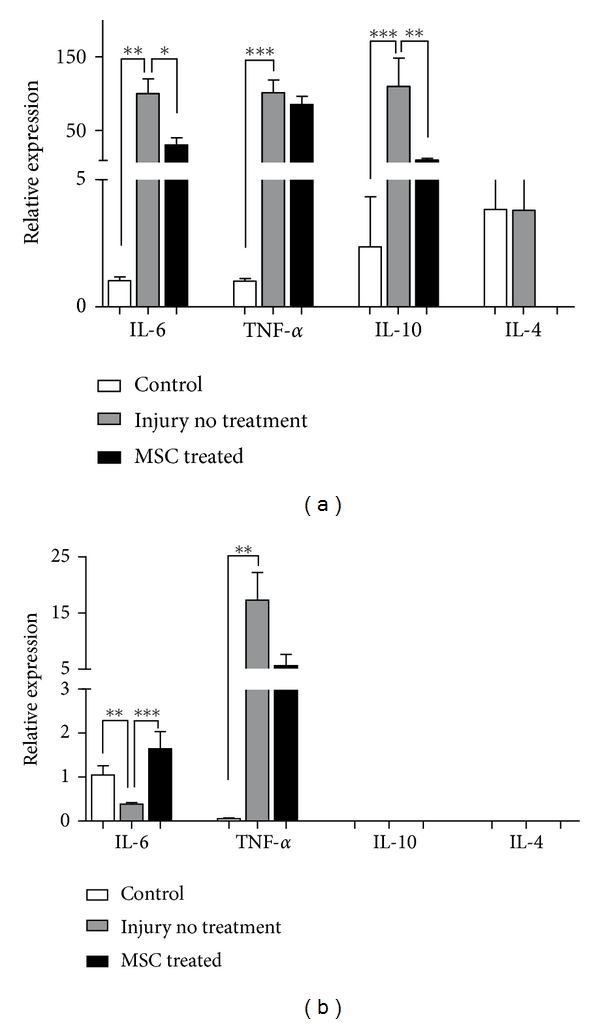
Relative expression of IL-6, TNF-*α*, IL-10, and IL-4 in acute and chronic models of motor cortex injury. (a) 24 hours and (b) 30 days after injury and MSC injection, RNA was extracted from motor cortex of control, injury without treatment and MSC treated mice. qPCR was performed to quantify the expression of inflammatory cytokines and relative expression was calculated in relation to HPRT. Data are expressed as mean of 2^−ΔΔCt^  ± SEM (control *n* = 3, injury no treatment *n* = 6, MSC treated *n* = 4). *P < .05, **P < .001, ***P < .0001.

Acute TBI results in upregulation of anti- and proinflammatory cytokines expression 24 hours after the injury (Figure 1(a)). Thirty days after injury, the expression levels of IL-10 and IL-4 are undetected whereas the expression of TNF-*α* persists significantly higher than in undamaged tissue and IL-6 expression decreases (Figure 1(b)). 

Our results show that immunomodulation by MSC transplantation in the TBI acute model is indicated by downregulation of IL-6, and IL-10 mRNA levels (Figure 1(a)). Thirty days after injury and injection of MSC, we observed increased expression of IL-6 (Figure 1(b)). 

### 3.2. Transplanted MSC also Modulate Serum Levels of Cytokines

**Figure 2:**
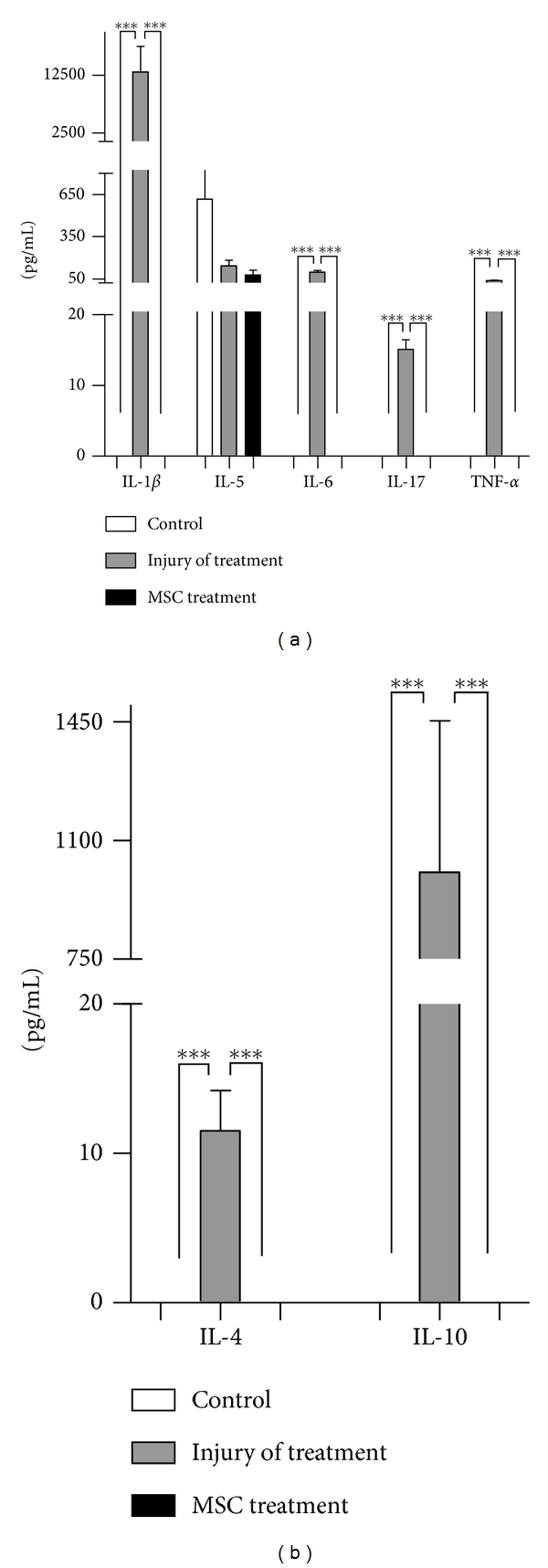
Cytokine profile in serum in TBI acute phase. Serum from mice submitted to the TBI protocol was used to investigate for cytokines content. Mice were transplanted (MSC treated) or not (injury not treated) with MSC immediately after the injury and blood was collected after 24 hours. (a) Pro-inflammatory cytokines and (b) anti-inflammatory cytokines (control *n* = 3, injury no treatment *n* = 6, MSC treated *n* = 4). ***P < .001, **P < .01.

TBI induces an inflammatory reaction by the release of proinflammatory cytokines IL-1*β*, IL-6, and IL-8 that can be detected in the serum of patients that have suffered severe TBI [32, 33]. There is very limited information about serum levels of cytokines in TBI model we used, especially regarding the levels of the pro-inflammatory interleukins seen in patients. Similar to what is observed for humans, the serum level of four pro-inflammatory cytokines, IL-1*β*, IL-6, IL-17, and TNF-*α* was increased by the injury (Figure 2(a)). We also observed increased levels of two anti-inflammatory cytokines, IL-4 and IL-10 (Figure 2(b)). Interestingly, MSC transplantation into the injury site decreased the serum levels of all cytokines in the acute phase of the model (Figure 2). 

**Figure 3:**
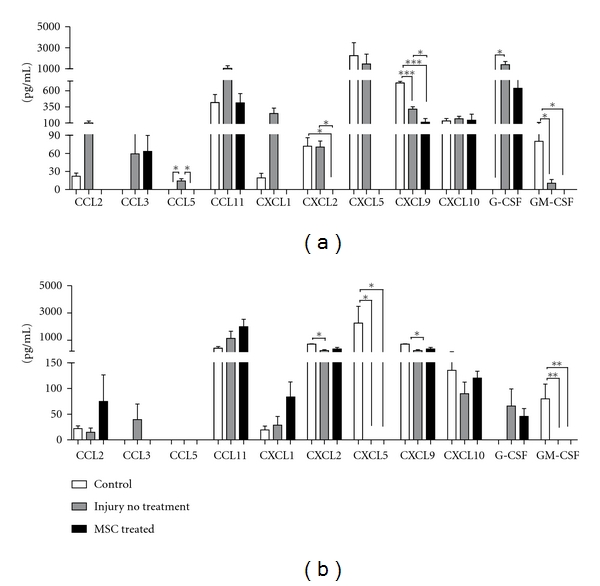
Chemokine profile in serum in TBI acute and chronic phase. Serum from mice submitted to the TBI protocol was used to investigate for cytokines content. Mice were transplanted (MSC treated) or not (injury not treated) with MSC immediately after the injury and blood was collected after 24 hours (acute phase) (a) or 30 days (chronic phase) (b). (control *n* = 3, injury no treatment *n* = 6, MSC treated *n* = 4). *P < .005, ***P < .001.

We expanded the investigation of the levels of cytokines to several chemokines, because of their important role in the modulation of the activity of immune cells, as well as of stem cells, especially regarding mobilization of stem cells from tissue-specific stem cell niches [34, 35]. We observed a variety of responses to injury and to MSC transplantation in the acute (24 hours) and chronic (30 days) phases. CCL5, CXCL9, and CXCL10 serum levels did not change in response to injury or to MSC transplantation after injury, in both acute and chronic phases (Figure 3). CCL2, CCL3, CCL11, CXCL1, and G-CSF serum levels increased 24 hours after injury in nontreated animals (Figure 3(a)) whereas only CCL2 and CCL11 returned to control levels after 30 days, even without MSC transplantation (Figure 3(b)). GM-CSF level was lower 24 hours after injury when compared to serum levels from control animals (Figure 3(a)), and was undetected after 30 days (Figure 3(b)). 

MSC transplantation decreased serum levels of CCL2, CCL11, CXCL1, CXCL5, CXCL9, CXCL2, and GM-CSF to normal, or even below normal values, 24 hours after injury. On the other hand, analysis of serum of animals that received MSCs 30 days after injury and transplantation showed that the levels of CCL2, CCL11, and CXCL1 were higher than the levels found in normal animals serum (Figure 3). 

### 3.3. MSC-Conditioned Medium Stimulates Proliferation of Neural Stem Cells and Modulates Gene Expression In Vitro

Many reports describe that soluble factors secreted at brain injury sites, including chemokines and cytokines, induce neural stem cells to migrate towards the injury site [36–38]. A recent study from our laboratory (unpublished data) showed that peptides analogous to the chemokine CXCL12, known to chemoattract cells that express the receptor CXCR4 such as lymphocytes, progenitor cells, hematopoietic, neural, and embryonic cells [39], stimulated chemotaxis of neuroblasts towards a cortex injury site, and modulated gene expression *in vivo*. Nevertheless, the hostile environment created by the inflammatory mediators secreted by glial and immune cells that infiltrate the injured brain reduces the survival of the migrating neuroblasts. 

**Figure 4:**
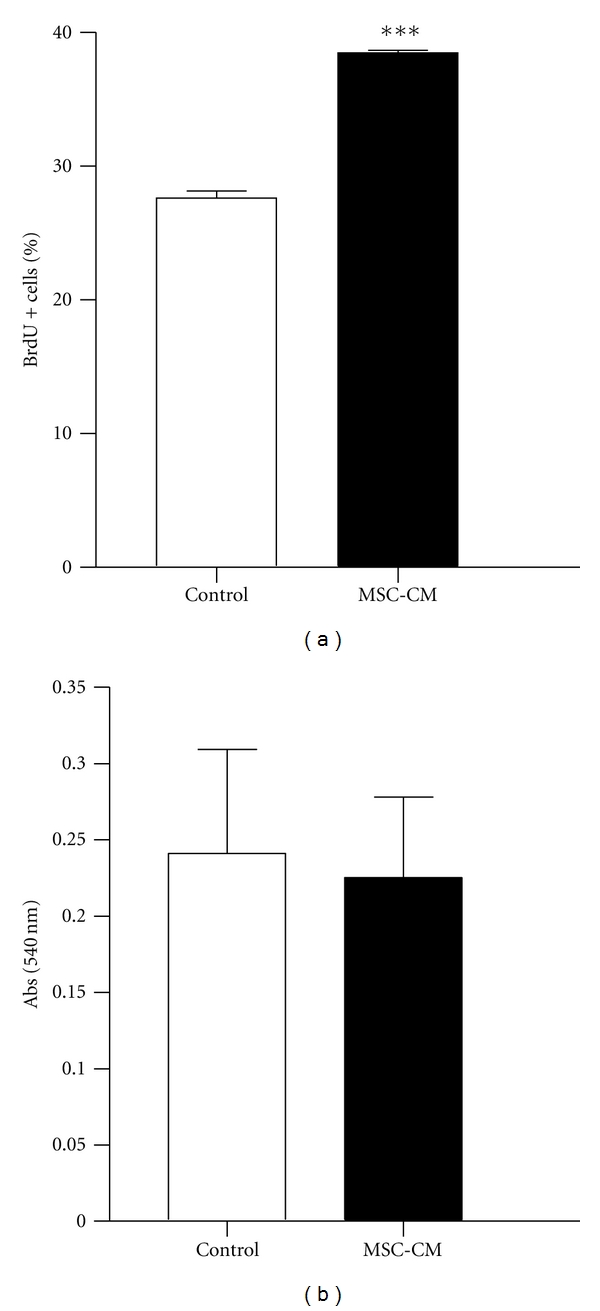
MSC secreted factors stimulate adult NSC proliferation. (a) Survival of NSC measured by MTT assay. (b) Proliferation of NSC measured by BrdU incorporation using flow cytometry. Data are mean ± SEM of two independent experiments performed in triplicates. ***P < .001.

The *in vivo *effects of MSC immunomodulation that we observed in the TBI model lead us to ask whether MSC secrete factors that could modulate neural stem cells survival, proliferation, and differentiation*. In vitro *treatment of neural stem cells derived from adult mice SVZ, cultured as neurospheres, with MSC-conditioned medium (MSC-CM) resulted in increased proliferation (Figure 4(a)) without affecting survival of the cells (Figure 4(b)). 

**Figure 5:**
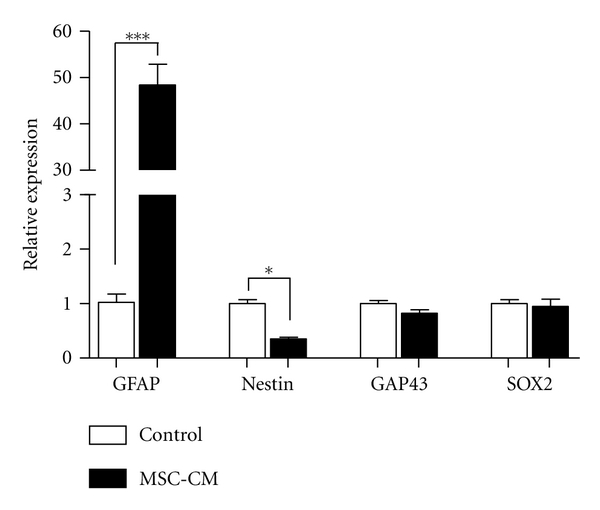
MSC secreted factors upregulate GFAP expression by adult NSC. RNA was extracted from neurospheres cultured in control medium or MSC-CM, and qPCR was performed to evaluate the expression of GFAP, nestin, GAP43, and SOX2. Relative expression was calculated in relation to GAPDH. Data expressed as mean of 2^−ΔΔCt^  ± SEM of triplicates. *P < .05, ***P < .0001.

Soluble factors present in MSC-CM changed neural stem cell gene expression, inducing upregulation of GFAP, a marker of astrocytes intermediate filaments, and downregulation of nestin, a marker of immature cells (Figure 5). The expression levels of GAP43, an axonal growth marker, and SOX2, a neural stemness marker, did not change (Figure 5). 

## 4. Discussion

Recently, the immunomodulatory properties of MSC have been investigated in a number of pathological situations. Despite MSC multipotency and self-renewing characteristics, studies suggest that the tissue regenerative potential exerted by these cells are not due to transdifferentiation and substitution of dead cells, but is due to the secretion of soluble factors which stimulate local progenitor cells to survive, proliferate, and differentiate [40, 41]. Several studies have demonstrated this effect in different pathologies, such as acute kidney injury [42], chronic renal failure [43], renal fibrosis [44], myocardium infarction [45], asthma [46], multiple sclerosis and amyotrophic lateral sclerosis [47], and brain ischemia [48]. In the present study, we used a murine TBI model performed in the motor cortex of mice. 

Our results show that 24 hours after the injury there is a significant increase in the expression of the pro-inflammatory cytokines IL-6, and TNF-*α* in the injury site (Figure 1). The increase in pro- and anti-inflammatory mediators, such as IL-6, and IL-10, have been described in cerebrospinal fluid as well as in serum of patients with severe TBI [49]. 

In the model we used, MSC transplantation significantly decreased the expression of IL-6, and similar results were observed regarding the anti-inflammatory cytokines IL-10 and IL-4, both locally and in serum (Figures 1 and 2). 

IL-6 is a pleiotropic cytokine, involved in several pathological situations and can play pro- or anti-inflammatory effects depending on the microenvironment [50]. Our results show that there was an increase in IL-6 expression at the injury site and also in serum (Figure 2). IL-6 is secreted mainly by microglia and astrocytes, and its neuroprotector role is characterized by TNF-*α* inhibition and NGF stimulation. IL-6 also promotes tissue revascularization and tissue scar formation [4]. Our results show increased IL-6 levels 30 days after injury and MSC transplantation, contrary to its decrease in the acute phase of the injury (24 hours after injury). These results suggest the immunomodulatory property of MSC in the TBI model where in a hostile environment in the acute period, when IL-6 is deleterious to the tissue, MSC downregulates IL-6, while after a few days, MSC upregulates IL-6, in a period when this chemokine would become beneficial to a possible tissue regeneration. Besides the alterations in cytokines expressions in the site of injury, MSC transplantation also influenced the levels of chemokines systemically, both in the acute (24 hours) and chronic (30 days) phases of TBI (Figure 3). It has been described that administration of IL-1*β* to the brain induces hepatic CXC and CCL chemokine synthesis, which correlates to elevation of circulating leukocytes in the blood [51]. 

CNS injury involves glial activation, leukocytes recruitment, increase in the expression, and secretion of inflammatory mediators as cytokines, and chemokines [6]. Recent studies have described molecular signals regulating NSC migration in the injured brain, including angiogenic factors, chemokines, cytokines and extracellular matrix components [52]. Brain injury induces proliferation of NSC in the SVZ and migration of neuroblasts from the SVZ towards the site of the insult [53]. Nevertheless few neuroblasts arrive at the injury site and survive to the injury microenvironment [12]. 

Our aim was to verify if the immunomodulatory properties observed in the *in vivo *experiments by the secretion of soluble factors by MSC could have any direct effect on NSC survival, proliferation, and/or differentiation. In our experiments, exposing NSC to soluble factors present in MSC-CM did not influence NSC apoptosis (data not shown) and survival, but increased proliferation, indicating that the factors secreted by MSC may contribute to enhancing the number of NSC without affecting survival (Figure 4). 

On the other hand, MSC-CM soluble factors modulated NSC gene expression, upregulating GFAP expression, downregulation of nestin, and no alterations in GAP43 and SOX2 expression (Figure 5). SOX2 is a transcription factor expressed by NSC present in neurogenic niches and maintains pluripotency or stemness state [54]. Constitutive expression of SOX2 inhibits neuronal differentiation, so this could explain nestin downregulation. 

## 5. Conclusion

In this study we observed that MSC are able to modulate the inflammatory response in an acute TBI model by changing the expression of pro- and anti-inflammatory cytokines, along with modulation of serum levels of several chemokines. In addition, MSC secreted soluble factors are capable of stimulating proliferation of NSC *in vitro*, as well as increasing the expression of GFAP, a gene related to mature astrocytes, indicating that factors expressed by MSC could induce differentiation of NSC *in vivo*. Altogether these results indicate that there are still open possibilities of new regeneration therapies to treat nervous tissue injuries, highlighting the importance of immunomodulatory properties of MSC and its potential to allow NSC survival and proliferation.
